# Interactive Role of Surrogate Liver Fibrosis Assessment and Insulin Resistance on the Incidence of Major Cardiovascular Events

**DOI:** 10.3390/jcm11175190

**Published:** 2022-09-01

**Authors:** Diego Martinez-Urbistondo, Delia D’Avola, David Navarro-González, Laura Sanchez-Iñigo, Alejandro Fernandez-Montero, Nuria Perez-Diaz-del-Campo, Elisabetta Bugianesi, Jose Alfredo Martinez, Juan Carlos Pastrana

**Affiliations:** 1 Internal Medicine Department, Clínica Universidad de Navarra, 28027 Madrid, Spain; 2 Liver Unit, Clínica Universidad de Navarra, 28027 Madrid, Spain; 3 Centro de Investigación Biomédica en Red en Enfermedades Hepáticas y Digestivas (CIBERehd), 28027 Madrid, Spain; 4 Navarra Health Service—Osasunbidea, 31003 Pamplona, Spain; 5 Instituto de Investigación Sanitaria de Navarra (IdiSNA), 31008 Pamplona, Spain; 6 Department of Occupational Medicine, University of Navarra, 31008 Pamplona, Spain; 7 Department of Medical Sciences, University of Turin, 10124 Turin, Italy; 8 Precision Nutrition and Cardiometabolic Health Program, IMDEA—Food Institute (Madrid Institute for Advanced Studies), Campus of International Excellence (CEI) Universidad Autónoma de Madrid (UAM) + Consejo Superior de Investigaciones Científicas (CSIC), 28027 Madrid, Spain; 9 Centro de Investigacion Biomedica en Red Area de Fisiologia de la Obesidad y la Nutricion (CIBEROBN), 28027 Madrid, Spain

**Keywords:** MAFLD, FIB4, TyG, MACE, cardiovascular risk

## Abstract

**Introduction**: The combination of easy-to-obtain validated biomarkers is interesting in the prognostic evaluation of patients at cardiovascular risk in a precision medicine scenario. The evaluation of the effect modification of insulin resistance and liver fibrosis with the Triglyceride-Glucose index (TyG) and Fibrosis-4 index (FIB4) might provide prognostic information in patients at cardiovascular risk. **Patients and methods:** A retrospective cohort study was performed with 2055 patients recruited in the Vascular Metabolic CUN cohort. The studied outcome was the incidence rate of major cardiovascular events (MACE). The Systematic Coronary Risk Evaluation (SCORE), FIB4 and TyG indexes were calculated according to validated formulas. **Results:** FIB4 and TyG showed a synergistic interaction using validated cut-offs for both indexes in the prediction of MACE (Hazard ratio (HR) 1.05 CI95% 1.01–1.08) which remained after adjustment by age, sex, SCORE subgroup, presence of diabetes, or previous MACE using standardized cut-off (HR 2.29 CI95% 1.33–3.94). Finally, a subgroup with significant TyG and FIB4 showed a higher cardiovascular risk in the study population (adjusted HR 3.34 CI 95% 1.94–5.77). **Conclusion:** The combined interpretation of TyG and FIB4 indexes might have a potential predictive value of major cardiovascular events.

## 1. Introduction

Metabolic associated fatty liver disease (MAFLD), previously known as non-alcoholic fatty liver disease (NAFLD), affects more than 25% of the adult population [1]. The elevated rates of this condition have been accompanied by an increasing prevalence of other non-communicable illnesses, such as cardiovascular and advanced liver diseases, with direct impact on patient co-morbidity and premature mortality [2,3,4,5]. Among them, cardiovascular disease is the leading cause of death in patients with MAFLD [6,7]. Indeed, cardiometabolic risk in these patients seems to strongly rely on the presence of advanced stages of MAFLD, including nonalcoholic steatohepatitis (NASH) and advanced liver fibrosis [6,7].

Insulin resistance and liver fibrosis have demonstrated complex interlinked connections affecting cardiovascular damage evolution [8,9,10]. Thus, insulin resistance is associated to the progression of liver fibrosis by promoting profibrogenic metabolic pathways and enhancing intrahepatic inflammation through activation of hepatic stellate cells [8,9] among others. On the other hand, liver fibrosis reduces the hepatic homeostatic capacity to counteract the deleterious effect of insulin resistance, hyperglycemia, and hypertriglyceridemia [10]. Moreover, both mechanisms have been described as clinically relevant and potentially synergistic in the prediction of cardiovascular risk within the metabolic syndrome setting [11].

Cardiovascular risk should be assessed using available and easy to interpret biomarkers. In this context, widespread tools such as the Systematic Coronary Risk Evaluation (SCORE) index that includes age, sex, smoking status, total cholesterol, and systolic blood pressure are able to provide a 10-year risk measure of major adverse cardiovascular event (MACE) incidence [12]. Subsequently, other variables such as the presence of type 2 diabetes or previous cardiovascular disease have been added to the SCORE analysis to provide therapeutic targets of LDL depending on longitudinal risk [13]. The inclusion of pathological concepts such as insulin resistance or liver fibrosis in these predictive scales is hampered by the absence of proven robust widespread biomarkers.

These issues are being progressively solved with the inclusion of newer combinations of routine use biomarkers which provide an acceptable correlation to the gold standard [14]. In the insulin resistance scenario, different combinations of plasma lipids and glucose have been proven to correlate to HOMA index in the prediction of insulin resistance [15]. Among them, the triglyceride and glucose index (TyG) is a strong predictor of liver steatosis, diabetes, metabolic syndrome, and cardiovascular events, being reproduced in different populations [16,17,18].

Furthermore, liver fibrosis non-invasive assessment has been recently empowered, with the development of new scores for the validation of previous scales in MAFLD [19]. Foremost among them, the Fibrosis-4 index (FIB4) has been included as a potential non-invasive screening proxy for fibrosis by the European Association for the Study of the Liver (EASL) [14]. Furthermore, this index has demonstrated a prognostic value in the incidence of different cardiovascular events [20,21,22].

Finally, accumulating evidence from the most recent pharmacological clinical trials in patients with MAFLD suggests a broad spectrum of cardiometabolic potential targets with a direct impact on personalized cardiovascular risk prevention [23]. In this context, the study of the putative interaction between liver fibrosis and insulin resistance, using easy to use and universally validate scores, might help in the universal individualization of cardiovascular risk assessment and treatment tailoring. Thus, the objective of this study is to evaluate and analyze the combination of a two minimally invasive surrogate markers of liver fibrosis and insulin resistance in the prediction of major cardiovascular events.

## 2. Patients and Methods

### 2.1. Study Design

The Vascular Metabolic CUN cohort is a population-based, retrospective epidemiological study designed to examine the incidence of cardiovascular and metabolic diseases including type 2 diabetes, hypertension, obesity, stroke, or coronary heart disease in a large Southern European population, recording patients from the 1 February 1997 to the 31 December 2002, and subsequently followed up until 31 December 2012. The cohort has been described elsewhere [24]. Briefly, exclusion criteria were age <18 or >90 years, history of type 1 diabetes or latent autoimmune diabetes of the adult, cancer in the palliative phase, previously diagnosed liver disease, familial hypertriglyceridemia, extreme body mass index (BMI) (>45 kg/m^2^), or an inherited and clinically relevant hypercoagulable state.

The present study included patients aged between 50 and 75 years from this cohort as considered ages in which cardiovascular risk assessment has a high impact on disease prevention. Patients with missing variables concerning age, sex, hypertension, and parameters concerning cardiovascular co-morbidities, the calculation of SCORE, TyG or FIB-4 indexes were excluded. The research was conducted according to the standards of the Declaration of Helsinki on medical research and was approved by the Ethics Committee of the University of Navarra (30/2015).

### 2.2. Covariables

Data regarding medical history, health-related behaviors, and blood biochemical measurements were retrieved at each patients’ visit. Health-related behaviors including cigarette smoking (none, former smoker, or current smoker), daily alcohol intake (yes/no), and lifestyle pattern (physically active/sedentary behavior) were obtained by physicians at the consultation. Before the measurement of blood pressure (BP), subjects waited for 5 min in a seated position. The BP on the indistinctly right or left arm was measured twice and the average value was recorded following World Health Organization criteria. Hypertension was defined based on the World Health Organization–International Society of Hypertension Guidelines as ≥140 (systolic BP)/90 (diastolic BP) mm Hg or when the subjects reported use of antihypertensive medication [25]. Cardiovascular disease was defined according to the International Classification of Diseases Tenth Revision (ICD-10) [26]. Anthropometric measurements and determinations of biochemical parameters including fasting plasma glucose (FPG), total cholesterol (TC), triglycerides (TG), HDL-cholesterol (HDL-C), LDL-cholesterol (LDL-C), neutrophil and lymphocyte count, and liver enzymes (ASAT and ALAT) were obtained and analyzed as described elsewhere [23]. TG was measured using enzymatic colorimetric tests and LDL-C was calculated using the Friedewald formula. The values of LDL-C were considered missing in patients with TG levels >400 mg/dL [27].

### 2.3. Main Variables

The SCORE index was calculated according to a previously validated formula [12] and patients were categorized into low, moderate, and high risk according to SCORE risk and previous morbidity according to the lipid management practice guidelines [13]. Due to the retrospective recruitment, patients with diagnosed type 2 diabetes mellitus and no other comorbidity with no information about diabetes onset (*n* = 6) were considered as high-risk patients to avoid bias.

The TyG index was calculated as previously described [27,28].

TyG index = ln[fasting TG (mg/dL) × FPG (mg/dL)/2]

A cut-off of 8.8 points was used to determine patients with severe insulin resistance risk and high probability of metabolic syndrome development. This cut-off was chosen due to the high previously described insulin resistance of the cohort and using previously validated studies in a Hispanic population [18].

The hepatic steatosis index (HSI) was calculated according to previous publications, using HIS > 36 as a cut-off high risk of significant infiltration of the liver by fat [29]. The FIB4 index was calculated using the following formula [30]:

$$\mathrm{FIB}-4\;\mathrm{index}=\frac{\mathrm{Age}\left(\mathrm{years}\right)\times \mathrm{AST}\left(\mathrm{UI}/L\right)}{\mathrm{Plateletcount}\left({\mathrm{mm}}^{3}\right)\times \sqrt{\mathrm{GPT}\left(\mathrm{UI}/L\right)}}$$

Patients were considered at significant risk following the non-invasive assessment of liver fibrosis in screening proposed by the EASL when values of FIB4 were >1.3 points [14].

The incidence of MACE was defined by the ICD-10 code list [25] from two subgroups: Coronary heart disease including codes from I20 to I25 and cerebrovascular disease including codes from I63 to I66.

### 2.4. Statistical Analyses

Categorical variables were presented as percentages. Student’s *t*-test, one-way ANOVA, or X^2^ test were implemented to compare the baseline characteristics of study participants. Different Cox proportional-hazard analysis to estimate the univariate hazard ratio (HR) and their 95% confidence interval (CI) of MACE of different variables with potential capacity to personalize cardiovascular risk were fitted. Further progressive Cox models were developed to evaluate the effect and interactions of TyG and FIB4 index in the prediction of MACE. After demonstration of interaction between TyG and FIB4 using previously described cut-off values, patients were divided in four clinical subgroups: (i) “Healthy subgroup” including patients with TyG index <8.8 and FIB4 index <1.3, (ii) “Liver fibrosis subgroup” including patients with TyG index < 8.8 but FIB4 index >1.3, (iii) “Insulin resistance subgroup” including patients with TyG index >8.8 and FIB4 index <1.3 and (iv) “Liver fibrosis and insulin resistance subgroup” including patients with TyG index >8.8 and FIB4 index >1.3. All statistical analyses were performed with SPSS version 20 (IBM Corp. Released 2011. IBM SPSS Statistics for Windows, Version 20.0. Armonk, NY, USA: IBM Corp). All *p*-values are two-tailed and statistical significance was set at the conventional cut-off of *p* < 0.05.

## 3. Results

A total of 2055 patients met the inclusion criteria and made up the study cohort. In the analysis of this cohort, a higher cardiovascular risk category according to the SCORE index was associated to older age and higher BMI (*p* < 0.01). Patients with higher CV risk were more likely to be male and have diagnosed hypertension, diabetes mellitus, and previous cardiovascular events (*p* < 0.01). In addition, patients at higher risk were also more likely to have higher values of systolic and diastolic blood pressure, total cholesterol, triglycerides, fasting glucose, TyG index values, neutrophil to lymphocyte ratio (NLR) values, HSI values, and FIB4 values (*p* < 0.01) as shown (Table 1).

**Figure 1:**
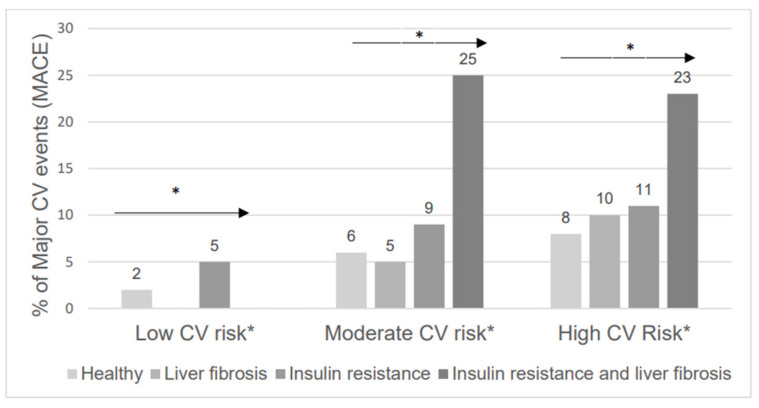
Major adverse cardiovascular events distribution according to liver fibrosis status and insulin resistance among CV risk subgroups. * *p* for trend < 0.01.

In the prediction of incident MACE different biomarkers of cardiovascular risk were evaluated with univariate Cox regression analysis. Only TyG index and FIB4 index demonstrated a consistent trend towards incidence of MACE in some of the CV risk subgroups (*p* < 0.10) as opposed to BMI, LDL levels, NLR, and HSI values, which remained far from statistical significance (*p* > 0.20) as shown (Table 2). Additional univariate analyses were performed to evaluate the distribution of different cardiovascular risk factors in the FIB4, TyG and FIB4, and TyG subgroups (Supplementary Tables S1–S3). The combination of significant liver fibrosis (FIB4 > 1.3) and significant insulin resistance at metabolic syndrome levels (TyG > 8.8) provided a synergistic effect on cardiovascular risk prediction, with a risk of MACE > 20% in the global cohort and in both moderate and high-risk subgroups when analyzed separately (*p* < 0.01) which elucidated the potential interaction between both concepts (Figure 1).

Then, a progressively adjusted Cox regression analysis was performed. All models were adjusted by SCORE as used in the adjustment of lipid control management [14], including age, sex, cardiovascular morbidity, previous diagnosis of DM, systolic blood pressure, and smoking status. In Model 1, FIB4 and TyG were evaluated as continuous variables. The TyG index (HR 1.71 CI 95% 1.30–2.24) as well as FIB4 (HR 1.48 CI 95% 1.07–2.04) showed a significant, independent, and linear association to MACE incidence. The further evaluation of the interaction between TyG and FIB4 indexes as continuous variables provided a statistically significant result for the interaction (HR = 1.05 1.01–1.08) in Model 2. In Model 3, the interaction between TyG and FIB4 evaluated as categorical values (TyG > 8.8 and FIB4 > 1.3) provided a HR of 2.57 (CI 95% 1.52–4.34), which was sustained after age and sex adjustment (HR 2.12 CI 95% 1.24–3.64) in Model 4 and after age, sex, diabetes, or cardiovascular events at baseline (HR 2.29 CI 95% 1.33–3.94) in Model 5.

**Figure 2:**
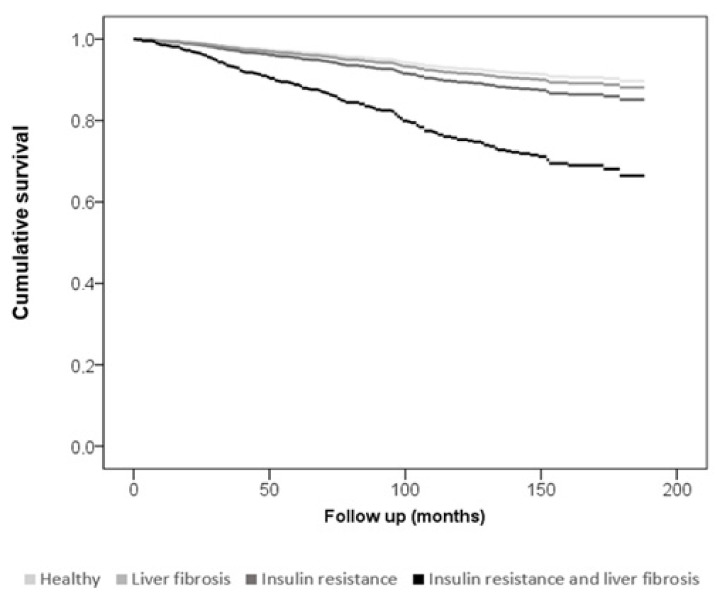
Kaplan–Meier graph for Cox regression analysis of the combination of insulin resistance and liver fibrosis in the prediction of MACE adjusted by cardiovascular risk (Log rank < 0.001).

Finally, in Model 6, patients were categorized in 4 subgroups: (i) “Healthy subgroup” including patients with TyG index < 8.8 and FIB4 index < 1.3, (ii) “Liver fibrosis subgroup” including patients with TyG index < 8.8 but FIB4 index > 1.3, (iii) “Insulin resistance subgroup” including patients with TyG index > 8.8 and FIB4 index < 1.3 and (iv) “Liver fibrosis and insulin resistance subgroup” including patients with TyG index > 8.8 and FIB4 index > 1.3 (Table 3) and represented in an adjusted Kaplan–Meier plot adjusted by CV risk for Cox regression analysis in which the last group outcome outperformed the rest in accumulated survival (HR 3.34 CI 95% 1.94–5.77) as shown (Figure 2). Additional models were performed to include other biomarkers, such as NLR, but no statistically significant results were found.

## 4. Discussion

The outcomes from the current investigation highlight that the combined evaluation of insulin resistance and liver fibrosis with minimally invasive biomarkers may represent a useful tool for the prediction of MACE independently of others cardiovascular risk assessment scores. In fact, the combination of the TyG index and the FIB4 index, both widely validated surrogate markers of insulin resistance in metabolic syndrome—liver steatosis and liver fibrosis, respectively—might help to identify a subgroup of patients with moderate and high cardiovascular risk according to SCORE, which may have a further risk of CV events (>20% of absolute risk of MACE), even within the same SCORE categories. These results might be explained due to a synergistic effect between insulin resistance and liver fibrosis denoted by the interaction between TyG > 8.8 points and FIB4 > 1.3 points (HR = 3.34, *p* < 0,01).

The present cohort resembles other previously described and distributed through cardiovascular disease risk subgroups [13]. In this context, patients with higher cardiovascular risk had worse prognostic features, being older, more frequently men and overweight, with a heavier morbidity burden and worse metabolic profile as assessed by commonly used biomarkers (FPG, triglycerides, TC, and LDL-C and HDL-C). In addition, MACE incidence increases progressively along risk groups. Although a higher than expected proportion of events according to SCORE probability was found in this series, the retrospective nature of this cohort and the historic date of patient assessment may explain partly these results due to the progressive amelioration in cardiovascular risk prevention. The liver fibrosis distribution was also consistent with previously described cohorts [11], with a low percentage of patients with high risk of liver fibrosis (*n* = 19, 1%) and 19% of patients with potential liver fibrosis, with a FIB4 index above 1.3 points.

When evaluating different biomarkers, insulin resistance as measured with the TyG index and liver fibrosis assessment according to FIB4 remained consistent in the prediction of incident MACE. These findings mimic previously described cohorts in which the most important NAFLD factor in CV risk prediction was fibrosis in biopsy-proven individuals, in which liver fibrosis was demonstrated as an effective CV event predictor [31]. Hence, a meta-analysis of observational studies including 36 longitudinal cohorts demonstrated a linear relationship between CV risk and advanced fibrosis [32]. In this analysis, the presence of liver fibrosis acted as a moderator of effect of diabetes in these patients, assessed as a qualitative variable.

Nevertheless, although a coactive effect of plasmatic glucose excess and MAFLD has been previously published in large epidemiological cohorts [11,33], the findings of this study highlight the importance of the presence of insulin resistance in the pathological effect of liver fibrosis in the prediction of MACE and the capacity of surrogates of insulin resistance and liver fibrosis to interact in the prediction of cardiovascular disease. On the one hand, patients with elevated FIB-4 alone have no differences in cardiovascular risk with those with low FIB4 and low TyG (HR 1.00 CI 95% 0.63–1.58). On the other hand, patients with high TyG and high FIB4 had 1.5-fold a higher risk than those with high TyG alone and 3-fold higher CV disease than those with low TyG. The trend for statistical significance of the interaction between TyG and FIB4 values in linear models and the confirmation of the statistical positive interaction in the risk prediction of two validated cut-off values for both scores in non-diabetic patients point out a subgroup of patients with multiplicative cardiovascular risk, which might help clinical surveillance and might be included in the individual patient analysis if confirmed in further series. 

Moreover, this fact points toward the potential presence of underlying pathophysiological mechanisms explaining the synergistic effect between insulin resistance and liver fibrosis.

These processes have been proven in different research fields with different mechanistic approaches. Thus, in metabolomic studies, the fingerprint of MAFLD with an increase in specific lipid metabolites such as intrahepatic DAGs are the strongest predictors of insulin resistance, providing a lipidomic interaction between liver fat accumulation and insulin resistance in the progression of cardiovascular diseases [34]. Different molecular pathways have been described linking insulin resistance with advanced stages of MAFLD, including the activation of C-C chemokine receptors type 2 and 5 [35,36] or the upregulation of transcriptional coactivator with PDZ binding motif (TAZ) [37]. Among them, the Notchs activation might provide a singular explanation to the currently described phenomenon, contributing to the intrahepatic collagen deposition [38] with an increase of FoxO1 activation, promoting gluconeogenesis and aggravating cardiovascular risk [39]. Hence, genetic factors may also provide a pathophysiological link between insulin resistance and MAFLD progression such as the E167K variant of TM6SF2 [40]. In this arena, MBOAT7 suppression, a MAFLD progression-associated mechanism, could also be particularly implicated due to the downregulating effect of hyperinsulinemia over the gene expression, providing NAFLD progression and further cardiovascular damage [41].

In this context, the synergistic statistical effect of elevated FIB4 and TyG indexes could be explained partly regarding the time of exposure to insulin resistance. Therefore, patients with insulin resistance and fibrosis would have had an extended exposure to insulin resistance due to the previously reviewed mechanisms and consequently a higher cardiovascular risk [38,39,40,41]. Although the characteristics of the cohort do not allow to distinguish which of these phenomena explains the present effect, the results of the present study might be considered a cornerstone for further investigation in this area. In fact, the present research could highlight not only the importance of the inclusion of the interaction between insulin resistance and liver fibrosis in the evaluation of patients on a daily basis, but also the methodology to do so, using widespread and easy to obtain parameters to calculate indexes such as TyG and FIB4 capable of distinguishing subgroups at different cardiovascular risk, despite age, sex, and traditionally evaluated variables included in the SCORE index, applying with previously validated cut-offs.

The present research has some limitations. The retrospective recollection of data and the absence of control on medical interventions may reduce the impact of these results. Furthermore, the recruitment of patients in a real-life scenario may allow the influence of uncontrolled facts in the accuracy of the present results. The lack of data regarding pharmacological therapies for metabolic alterations such as antidiabetics, hypolipemiant, etc. may introduce a further bias, since the effect of different drugs in protecting endothelia and reducing the progression of fibrosis has not been taken into account; however, the adjustments in the multivariate models were designed to reduce this possibility by including LDL targets and the presence of diabetes. It may be speculated that patients with a longer duration of diabetes, insulin resistance, or other metabolic factors before study inclusion could have higher FIB4 value as well an increased cardiovascular risk. The adjustment for age of every regression model may reduce but not completely eliminate this bias. Similarly, the absence of longitudinal surveillance before the incidence of MACE did not allow an evaluation of the metabolic control on prospective cardiovascular risk.

The inclusion of patients with previously diagnosed cardiovascular events could also bias the results, although prevalent cardiovascular events are considered in cardiovascular risk assessment guidelines and in the SCORE scale. Furthermore, models including adjustments by previously diagnosed cardiovascular events were included to provide further consistency to the results. The selection of patients with an age range between 50 and 75 years may help to avoid the influence of extreme ages in the results, while the adjustment by a well-validated CV score [13], the previous validation of both TyG index and FIB4 indexes, as well as the use of proven cut-offs in the analysis [18,28,29] may contribute to partially solving these queries. The use of a high and previously validated cut-off for TyG is justified by the high insulin resistance of the present cohort (8.4+/0.6 points), while different cut-offs should be demonstrated in prospective analysis to provide further consistency to the present results. Despite these potential limitations, the methodology of the study, with a non-negligible sample size and follow up, the use of precision and previously validated tools in the adjustment of models and research such as the SCORE, TyG, and FIB4 indexes, and the plausibility of the results with previously published evidence [41] provide validity to the present results.

Finally, the suggestion of a synergistic interaction between widely available surrogate markers of liver fibrosis and insulin resistance may provide a very interesting clinical instrument. This finding, if confirmed in prospective and large cohorts, might be useful in the personalization of treatment in cardiovascular risk, by pointing out a potentially shady very high cardiovascular risk subgroup of patients. In fact, the current results highlight the potential importance of the insulin resistance added to liver fibrosis in the cardiovascular disease pathogenesis. The non-invasive evaluation of this interaction could help to elucidate the adequate candidates for the future treatment of liver fibrosis. Furthermore, the validation of “easy to calculate and use” indexes concerning liver fibrosis and insulin resistance in the cardiovascular risk scenario might be a useful tool in the patient selection for liver fibrosis treatments [23,41] contributing to the widespread of precision medicine.

## 5. Conclusions

The TyG index, as a surrogate marker of insulin resistance, and the FIB4 index, as a proxy of liver fibrosis, show an interactive potential, with predictive value of major adverse cardiovascular events.

## Figures and Tables

**Table 1:** Population characteristics.

Variable	Low CV Risk <1% Risk *n* = 240	Moderate CV Risk 1–5% Risk *n* = 770	High CV Risk >5% Risk *n* = 1045	*p*
Age, years, mean ± SD	54 ± 3	55 ± 5	63 ± 6	<0.01
Sex, female %	212 (88%)	327 (42%)	295 (28%)	<0.01
SCORE, % ± SD	0.29 ± 0.28	2.5 ± 1.15	8.8 ± 0.15	<0.01
BMI, mean ± SD	26.1 ± 4.6	27.2 ± 4.9	28.0 ± 4.2	<0.01
BMI > 25 kg/m^2^, %	126 (52%)	553 (71%)	784 (74%)	<0.01
**Co-morbidities at baseline**				
Hypertension, %	24 (10%)	192 (24%)	494 (46%)	<0.01
Controlled LDL (CV group adjusted), %	45 (19%)	45 (6%)	3 (1%)	<0.01
Never smoker, %	153 (80%)	342 (55%)	296 (33%)	<0.01
Daily alcohol consumption, %	47 (5.8%)	261 (32%)	498 (62%)	<0.01
Diabetes mellitus, %	0	0	179 (17%)	<0.01
Previous cardiovascular events, %	0 (0%)	0 (0%)	186 (28%)	<0.01
**Cardiovascular risk markers**				
Systolic blood pressure	118 ± 14	129 ± 15	143 ± 54	<0.01
Diastolic blood pressure	75 ± 8	81 ± 9	84 ± 23	<0.01
Total cholesterol, mg/dL ± SD	218 ± 31	221 ± 30	242 ± 46	<0.01
c-LDL, mg/dL ± SD	139 ± 26	145 ± 27	165 ± 41	<0.01
HDL, mg/dL ± SD	62 ± 16	55 ± 15	53 ± 14	<0.01
Triglycerides, mg/dL ± SD	83 ± 45	101 ± 56	116 ± 72	<0.01
Fasting Glucose, mg/dL ± SD	92 ± 10	96 ± 12	108 ± 31	<0.01
**Insulin resistance**				
TyG index, mean ± SD	8.1 ± 0.5	8.3 ± 0.5	8.6 ± 0.6	<0.01
TyG index > 8.8, %	21 (9%)	141 (18%)	328 (31%)	<0.01
**Chronic inflammation**				
NLR, mean ± SD	1.94 ± 1.4	1.95 ± 0.9	2.12 ± 1.1	<0.01
NLR < 1.5 points, %	95 (40%)	253 (33%)	286 (27%)	<0.01
NLR 1.5–3 points, %	121 (50%)	441 (57%)	616 (59%)	
NLR > 3 points, %	24 (10%)	78 (10%)	143 (14%)	
**MAFLD—HSI index**				
Mean HSI, mean ± SD	37 ± 6	38 ± 6	39 ± 6	<0.01
Low risk, %	16 (7%)	49 (6%)	44 (5%)	<0.01
Indeterminate risk, %	94 (39%)	229 (30%)	301 (29%)	
High risk, %	130 (54%)	493 (64%)	700 (67%)	
**Liver fibrosis—FIB4 index**				
Mean FIB-4 index, mean ± SD	0.88 ± 0.39	1.05 ± 0.78	1.18 ± 0.83	<0.01
Low risk, %	220 (91%)	657 (85%)	769 (73%)	<0.01
Intermediate risk, %	18 (8%)	106 (4%)	254 (25%)	
High risk, %	2 (1%)	8 (1%)	22 (2%)	
**Outcomes**				
Surveillance (months) mean ± SD	104 ± 60	105 ± 60	92 ± 60	<0.01
Acute ischemic cardiopathy, %	2 (1%)	30 (4%)	65 (6%)	<0.01
Cerebral ischemic attack, %	3 (1%)	18 (2%)	43 (4%)	<0.01
Major adverse cardiovascular events (MACE), %	5 (2%)	47 (6%)	108 (10%)	<0.01

Abbreviations: SD—standard deviation, BMI—body mass index, LDL—low density lipoprotein, HDL—high density lipoprotein, TyG—triglycerides to glucose index, NLR—neutrophil to lymphocyte ratio, MAFLD—metabolic associated fatty liver disease; HIS—hepatic steatosis index; FIB4—fibrosis index 4; MACE—major cardiovascular events.

**Table 2:** Univariate Cox regression analysis of major adverse cardiovascular events of biomarkers among CV risk groups.

	Moderate CV Risk HR (95% CI)	*p*	High CV Risk HR (95% CI)	*p*
**Adiposity**				
BMI kg/m^2^	1.04 (0.97–1.12)	0.23	0.97 (0.92–1.01)	0.22
Overweight, BMI > 25 kg/m^2^	0.96 (0.52–1.80)	0.91	0.84 (0.55–1.26)	0.40
**Insulin resistance**				
TyG index, mean	1.43 (0.82–2.50)	0.19	1.76 (1.28–2.41)	<0.01
Insulin resistance (TyG > 8.8)	2.00 (1.07–3.73)	0.03	1.73 (1.18–2.55)	<0.01
**Cholesterol levels**				
LDL levels, mg/dL	1.00 (0.99–1.01)	0.92	1.00 (0.99–1.01)	0.89
Low LDL	1.48 (0.21–1.38)	0.45	-	-
**Chronic inflammation**				
NLR, mean ± SD	1.05 (0.76–1.45)	0.74	0.97 (0.80–1.18)	0.77
NLR < 1.5 points				
NLR 1.5–3 points	0.67 (0.36–1.23)	0.28	0.68 (0.45–1.04)	0.08
NLR > 3 points	1.48 (0.62–3.53)	0.29	0.81 (0.44–1.49)	0.51
**Alcohol**				
Alcohol consumption (yes/no)	1.39 (0.72–2.71)	0.38	1.05 (0.70–1.60)	0.77
**MAFLD (HSI index)**				
HSI, mean ± SD	1.03 (0.98–1.08)	0.18	0.99 (0.92–1.02)	0.56
High risk (HSI > 36), %	1.26 (0.69–2.33)	0.44	0.96 (0.65–1.43)	0.86
**Liver fibrosis (FIB4 index)**				
FIB-4 index, mean ± SD	1.11 (0.47–2.64)	0.86	1.49 (1.05–2.13)	0.03
Liver fibrosis risk (FIB-4 > 1.3), %	1.69 (0.81–3.50)	0.15	1.40 (0.93–2.09)	0.09

Abbreviations: SD—standard deviation, BMI—body mass index, LDL—low density lipoprotein, TyG—triglycerides to glucose index, NLR—neutrophil to lymphocyte ratio, MAFLD—metabolic associated fatty liver disease; HIS—hepatic steatosis index; FIB4—fibrosis index 4; MACE—major cardiovascular events; CV—cardiovascular.

**Table 3:** Multivariate analysis of the association between insulin resistance and non-invasive liver fibrosis assessment in the prediction of MACE.

Variable	HR (CI 95%)	*p*
**Model 1 linear** **Adjusted by SCORE subgroup**		
Non-invasive assessment of liver fibrosis (FIB-4)	1.48 (1.07–2.04)	0.01
Insulin resistance assessment (TyG index)	1.71 (1.30–2.24)	<0.01
**Model 2 interaction** **Adjusted by SCORE subgroup**		
Insulin resistance assessment (TyG index)	1.61 (1.23–2.12)	0.01
FIB-4 index x TyG index (interaction)	1.05 (1.01–1.08)	0.01
**Model 3** **Adjusted by SCORE categorical interaction**		
Insulin resistance assessment (TyG index)	1.42 (1.06–1.92)	0.02
FIB-4 index > 1.3 points x TyG index > 8.8 points (interaction)	2.57 (1.52–4.34)	<0.01
**Model 4** **Adjusted by SCORE subgroup, age and sex**		
Insulin resistance assessment (TyG index)	1.54 (1.14–2.09)	0.01
FIB-4 index > 1.3 points x TyG index > 8.8 points (interaction)	2.12 (1.24–3.64)	<0.01
**Model 5** **Adjusted by age, sex, SCORE, DM and previous CVE**		
Insulin resistance assessment (TyG index)	1.51 (1.10–2.10)	0.02
FIB-4 index > 1.3 points x TyG index > 8.8 points (interaction)	2.29 (1.33–3.94)	0.01
**Model 6** **Adjusted by age, sex, SCORE, DM and previous CVE**		
*Healthy individuals* FIB-4 < 1.3 points/TyG < 8.8 points	1	
*Liver fibrosis* FIB-4 > 1.3 points/TyG < 8.8 points	1.00 (0.63–1.58)	0.98
*Insulin resistance* FIB-4 < 1.3 points/TyG > 8.8 points	1.51 (1.01–2.28)	0.04
*Insulin resistance and liver fibrosis* FIB-4 > 1.3 points/TyG > 8.8 points	3.34 (1.94–5.77)	<0.01

## Supplementary Materials

The following supporting information can be downloaded at: https://www.mdpi.com/article/10.3390/jcm11175190/s1, Table S1: Population characteristics among FIB-4 subgroups. Table S2: Population characteristics among Tyg subgroups. Table S3: Population characteristics among TyG index and FIB-4 subgroups.

## Abbreviations

TyG: triglyceride to glucose; FIB4: fibrosis-4 index; MACE: major adverse cardiovascular event; SCORE: Systematic Coronary Risk Evaluation; HR: hazard ratio; MAFLD: metabolic fatty liver disease; NASH: nonalcoholic steatohepatitis; EASL: European Association for the Study of the Liver; BMI: body mass index; BP: blood pressure; ICD-10: International Classification of Diseases Tenth Revision; FPG: fasting plasma glucose; TC: total cholesterol; TG: triglycerides, HDL-C: HDL-cholesterol; LDL-C: LDL-cholesterol (LDL-C); HSI: hepatic steatosis index; CI: confidence interval.
