# Supplementary material for: Interactive Role of Surrogate Liver Fibrosis Assessment and Insulin Resistance on the Incidence of Major Cardiovascular Events
Source: J Clin Med. 2022 Sep 1;11(17):5190. doi: 10.3390/jcm11175190 (PMC9456724; doi:10.3390/jcm11175190)
Supplement: Supplementary file 1 [file jcm-11-05190-s001.zip › jcm-1827064-supplementary.pdf]

Table S1. Population characteristics among FIB-4 subgroups.

Variable	Low Fibrosis Risk FIB4 < 1.3 *n*= 1653	Indeterminate Fibrosis Risk FIB4 1.3–2.67 *n* = 383	High Fibrosis Risk FIB-4 > 2.67 *n* = 19	*p*
Age, years, mean ± SD	60 ± 6	65 ± 5	65 ± 7	$p < 0.01$
Sex, female %	693 (42 %)	129 (33 %)	9 (47 %)	0.01
SCORE, % ± SD	4.7 ± 8	7.5 ± 6	8.1 ± 12	$p < 0.01$
SCORE subgroups				$p < 0.01$
Low risk, %	220 (13 %)	18 (5 %)	2 (10 %)	
Moderate risk, %	660 (40 %)	108 (28 %)	2 (10 %)	
High risk, %	773 (47 %)	257 (67 %)	15 (80 %)	
BMI, mean ± SD	27.5 ± 4	27.4 ± 4	26.6 ± 4	0.53
BMI >25 kg/m2, %	1185 (72 %)	254 (66 %)	12 (63 %)	0.08
**Co-morbidities at baseline**				
Hypertension, %	533 (32 %)	164 (43 %)	7 (37 %)	$p < 0.01$
Controlled LDL (CV group adjusted), %	76 (5 %)	11 (3 %)	2 (10 %)	0.14
Never smoker, %	634 (47 %)	145 (47 %)	11 (61 %)	0.34
Daily alcohol consumption, %	647 (51 %)	145 (50 %)	7 (41 %)	0.56
Diabetes mellitus, %	128 (8 %)	45 (11 %)	1 (5 %)	0.04
Previous cardiovascular events, %	130 (8 %)	50 (13 %)	3 (15 %)	0.01
**Cardiovascular risk markers**				
Systolic blood pressure	134 ± 45	138 ± 20	138 ± 25	0.28
Diastolic blood pressure	82 ± 10	84 ± 36	80 ± 9	0.07
Total cholesterol, mg/dl ± SD	233 ± 41	225 ± 39	234 ± 55	0.02
c-LDL, mg/dl ± SD	156 ± 37	150 ± 35	155 ± 53	0.65
HDL, mg/dl ± SD	55 ± 15	55 ± 14	58 ± 21	0.02
Triglycerides, mg/dl ± SD	108±	98±	121±	0.01
Fasting Glucose, mg/dl ± SD	102±	101±	100±	0.81
**Insulin resistance**				
TyG index, mean ± SD	8.5 ± 0.6	8.4 ± 0.5	8.5 ± 0.7	0.03
TyG index > 8.8, %	403 (24 %)	75 (20 %)	5 (26 %)	0.13
**Chronic inflammation**				
NLR, mean ± SD	2.04 ± 1.06	2.03 ± 0.96	2.07 ± 0.89	0.97
NLR < 1.5 points, %	517 (31 %)	114 (30 %)	7 (36 %)	$p < 0.01$
NLR 1.5–3 points, %	946 (57 %)	215 (56 %)	10 (53 %)	
NLR > 3 points, %	190 (11 %)	54 (14 %)	2 (11 %)	
**MAFLD—HSI index**				
Mean HSI, mean ± SD	39.3 ± 6.2	37.0 ± 6.1	36.7 ± 5.3	0.01
Low risk, %	72 (4 %)	38 (10 %)	1 (5 %)	
Indeterminate risk, %	462 (28 %)	150 (39 %)	10 (53 %)	
High risk, %	1119 (68 %)	195 (51 %)	8 (42 %)	
**Liver fibrosis—FIB4 index**				
Mean FIB-4 index, mean ± SD	0.88 ± 0.21	1.64 ± 0.31	3.13 ± 0.31	0.01
**Outcomes**				
Surveillance (months) mean ± SD	101 ± 59	92 ± 59	79 ± 64	0.01
Major adverse cardiovascular events (MACE), %	115 (7 %)	43 (11 %)	2 (11 %)	0.02

Table S2. Population characteristics among TyG index subgroups.

Variable	Low Insulin R Risk TyG < 8.8 *n*= 1.572	High Insulin R Risk Fibrosis Risk TyG > 8.8 *n* = 483	*p*
Age, years, mean ± SD	61 ± 7	61 ± 6	$p < 0.01$
Sex, female %	724 (46 %)	107 (22 %)	$p < 0.01$
SCORE, % ± SD	4.8 ± 7.1	7.1± 11.1	$p < 0.01$
SCORE subgroups			$p < 0.01$
Low risk, %	219 (14 %)	21 (4 %)	
Moderate risk, %	633 (40 %)	137 (28 %)	
High risk, %	720 (46 %)	325 (67 %)	
BMI, mean ± SD	26.9 ± 4.1	29.4 ± 4.1	$p < 0.01$
BMI >25 kg/m2, %	1031 (66 %)	420 (87 %)	$p < 0.01$
**Co-morbidities at baseline**			
Hypertension, %	493 (31 %)	211 (44 %)	$p < 0.01$
Controlled LDL (CV group adjusted), %	72 (5 %)	17 (4 %)	0.31
Never smoker, %	658 (51 %)	132 (33 %)	$p < 0.01$
Daily alcohol consumption, %	576 (48 %)	223 (61 %)	$p < 0.01$
Diabetes mellitus, %	69 (4 %)	105 (22 %)	$p < 0.01$
Previous cardiovascular events, %	122 (8 %)	61 (12 %)	0.01
**Cardiovascular risk markers**			
Systolic blood pressure	133 ± 38	140 ± 51	$p < 0.01$
Diastolic blood pressure	82 ± 20	85 ± 11	$p < 0.01$
Total cholesterol, mg/dl ± SD	228 ± 39	243 ± 45	$p < 0.01$
c-LDL, mg/dl ± SD	154 ± 35	159 ± 41	$p < 0.01$
HDL, mg/dl ± SD	58 ± 15	47 ± 12	$p < 0.01$
Triglycerides, mg/dl ± SD	82 ± 28	184 ± 83	$p < 0.01$
Fasting Glucose, mg/dl ± SD	96 ± 13	119 ± 38	$p < 0.01$
**Insulin resistance**			
TyG index, mean ± SD	8.2 ± 0.35	9.2 ± 0.39	$p < 0.01$
**Chronic inflammation**			
NLR, mean ± SD	2.05 ±1.08	1.98 ± 0.89	0.17
NLR < 1.5 points, %	479 (30 %)	159 (32 %)	0.58
NLR 1.5–3 points, %	900 (57 %)	271 (56 %)	
NLR > 3 points, %	193 (12 %)	53 (11 %)	
**MAFLD—HSI index**			
Mean HSI, mean ± SD	37.8 ± 5.8	42.3 ± 6.2	$p < 0.01$
Low risk, %	101 (6 %)	10 (2 %)	
Indeterminate risk, %	560 (36 %)	62 (13 %)	
High risk, %	911 (58 %)	411 (85 %)	
**Liver fibrosis—FIB4 index**			
Mean FIB-4 index, mean ± SD	1.05 ± 0.43	1.00 ± 0.42	$p < 0.01$
Low risk, %	1250 (80 %)	403 (83 %)	0.13
Indeterminate risk, %	308 (19 %)	75 (16 %)	
High risk, %	14 (1 %)	5 (1 %)	
**Outcomes**			
Surveillance (months) mean ± SD	102 ± 60	90 ± 57	$p < 0.01$
Major adverse cardiovascular events (MACE), %	103 (7 %)	57 (12 %)	$p < 0.01$

Table S3. Population characteristics among TyG index and FIB-4 subgroups.

Variable	Low IR/Low FIB *n* = 1250	Significant FIB/Low IR *n* = 403	Low FIB /Significant IR *n* = 322	Significant IR & Significant FIB *n* = 80	*p*
Age, years, mean ± SD	60 ± 6	65 ± 6	60 ± 6	64 ± 6	$p < 0.01$
Sex, female %	602 (48 %)	122 (38 %)	91 (23 %)	16 (20 %)	$p < 0.01$
SCORE, % ± SD	4.2 ± 7.1	6.9 ± 6.2	6.4 ± 11.3	10.3 ± 9.2	$p < 0.01$
SCORE subgroups					$p < 0.01$
Low risk, %	200 (16%)	19 (6 %)	20 (5%)	1 (1 %)	
Moderate risk, %	535 (43 %)	98 (30 %	125 (31 %)	12 (15 %)	
High risk, %	515 (41 %)	205 (64 %)	258 (64 %)	67 (84 %)	
BMI, mean ± SD	27.0 ± 3.9	27.0 ± 4.5	29.5 ± 4.2	28.7 ± 3.5	$p < 0.01$
BMI >25 kg/m2, %	835 (67 %)	196 (70 %)	350 (87 %)	70 (87 %)	$p < 0.01$
**Co-morbidities at baseline**					
Hypertension, %	365 (29 %)	128 (40 %)	168 (42 %)	43 (54 %)	$p < 0.01$
Controlled LDL (CV group adjusted), %	60 (5 %)	12 (4 %)	16 (4 %)	1 (1 %)	0.40
Never smoker, %	527 (51 %)	131 (53 %)	107 (32 %)	25 (34 %)	$p < 0.01$
Daily alcohol consumption, %	469 (49 %)	107 (60 %)	178 (45 %)	45 (65 %)	$p < 0.01$
Diabetes mellitus, %	48 (4 %)	21 (6 %)	80 (20 %)	25 (31 %)	$p < 0.01$
Previous cardiovascular events, %	86 (7 %)	36 (11 %)	44 (11 %)	17 (21 %)	$p < 0.01$
**Cardiovascular risk markers**					
Systolic blood pressure	132 ± 41	136 ± 59	140 ± 44	145 ± 23	$p < 0.01$
Diastolic blood pressure	81 ± 9	84 ± 39	84 ± 11	84 ± 11	$p < 0.01$
Total cholesterol, mg/dl ± SD	230 ± 38	222 ± 39	244 ± 45	238 ± 43	$p < 0.01$
c-LDL, mg/dl ± SD	155 ± 35	149 ± 35	160 ± 41	146 ± 38	$p < 0.01$
HDL, mg/dl ± SD	58 ± 15	57 ± 14	47 ± 11	47 ± 12	$p < 0.01$
Triglycerides, mg/dl ± SD	83 ± 29	80 ± 24	186 ± 86	174 ± 66	$p < 0.01$
Fasting Glucose, mg/dl ± SD	95 ± 13	96 ± 14	119 ± 38	122 ± 39	$p < 0.01$
**Insulin resistance**					
TyG index, mean ± SD	8.2 ± 0.3	8.2 ± 0.3	9.2 ± 0.4	9.2 ± 0.3	$p < 0.01$
**Chronic inflammation**					
NLR, mean ± SD	2.06 ± 1.11	2.03 ± 0.94	1.97 ± 0.87	2.01 ± 1.00	0.55
**MAFLD—HSI index**					
Mean HSI, mean ± SD	38.2 ± 5.7	36.2 ± 5.7	43.8 ± 6.1	39.9 ± 6.3	$p < 0.01$
**Liver fibrosis—FIB4 index**					
Mean FIB-4 index, mean ± SD	0.88 ± 0.47	1.71 ± 0.45	0.85 ± 0.46	1.71 ± 0.53	$p < 0.01$
**Outcomes**					
Surveillance (months) mean ± SD	104 ± 59	95 ± 59	92 ± 57	78 ± 54	$p < 0.01$
Major adverse cardiovascular events (MACE), %	77 (6 %)	26 (8 %)	38 (9 %)	19 (24 %)	$p < 0.01$
